# Clear health benefits for cleaner air

**DOI:** 10.1038/s43856-021-00049-5

**Published:** 2021-11-17

**Authors:** Ben Abbott

**Affiliations:** Communications Medicine, https://www.nature.com/commsmed

## Abstract

Air pollution is a major cause of poor health and premature death, but evidence on the impact of changing levels of air pollution on mortality rates is scarce. A recent study in *The BMJ* shows that relocating to an area with improved air quality is associated with decreased mortality.

**Figure:**
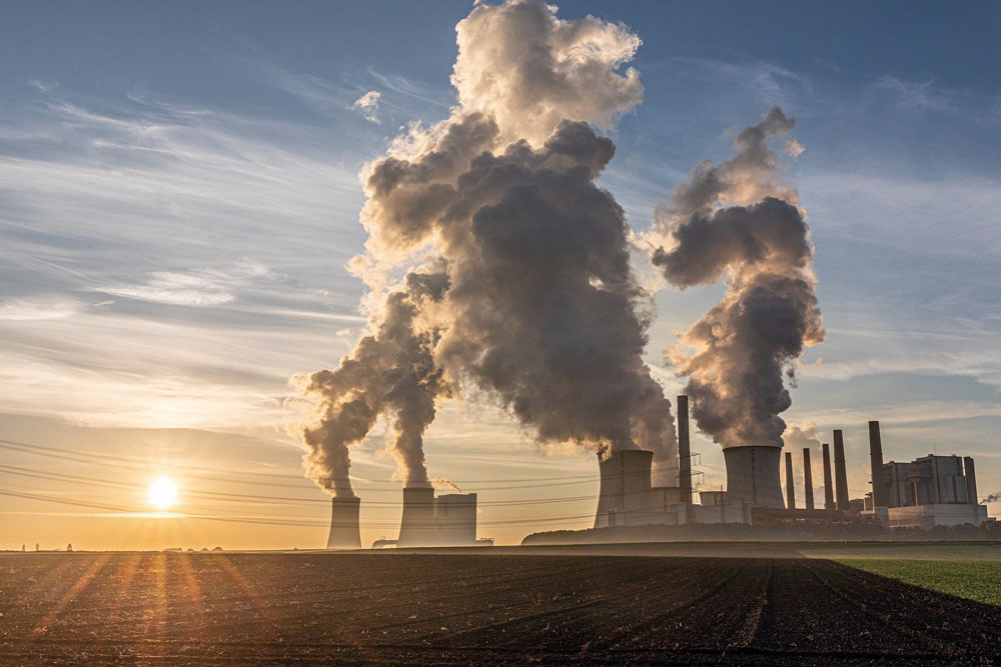
Pixabay

Exposure to air pollution is a major cause of cardiorespiratory disease and death, largely due to inhalation of ambient particulate matter less than 2.5 μm in diameter (PM_2.5_). Sources of fine particles of this size include the combustion of fuels in industrial and residential settings, as well as emissions from road vehicles.

While associations have been drawn between PM_2.5_ exposure and adverse health outcomes, including mortality, evidence to show that reducing exposure to PM_2.5_ has a beneficial effect is more limited. There are ethical implications of trying to address this hypothesis experimentally, given that a control group would need to be exposed to higher levels of PM_2.5_.

To circumvent this issue, Chen and colleagues used a quasi-experimental design in which they studied a sample of over 400,000 Canadians drawn from the Canadian Census Health and Environment Cohort who, over the period studied, had relocated to an area with lower or higher PM_2.5_ levels^1^. As such, it was possible to determine the impact of changes in residential PM_2.5_ exposure on deaths without researcher intervention.

Participants were grouped into three groups including those moving into areas with high, intermediate or low PM_2.5_ levels. Those who moved from an area with high PM_2.5_ levels to an area with intermediate or low levels had a 6.8% or 12.8% reduction in mortality, respectively, compared to participants who moved from an area with high PM_2.5_ levels to another area with high levels. Conversely, mortality increased by 1.8% in those who moved from an area with low PM_2.5_ levels to an area with intermediate levels and by 13.2% in those moving from a low to a high PM_2.5_ area. Decreases in PM_2.5_ levels were most strongly protective against deaths from cardiometabolic causes and increases in PM_2.5_ levels were mostly associated with deaths from respiratory causes.

While some important covariables were accounted for, such as socioeconomic status and comorbidities, data on others such as smoking status and neighbourhood-level deprivation were not available. Further studies will also be needed to determine the magnitude of the effect in areas with higher levels of air pollution than Canada. However, this large population-based study provides strong evidence in support of efforts to improve air quality and the positive effects these might yield on population health.
